# Prognostic Immunity and Therapeutic Sensitivity Analyses Based on Differential Genomic Instability-Associated LncRNAs in Left- and Right-Sided Colon Adenocarcinoma

**DOI:** 10.3389/fmolb.2021.668888

**Published:** 2021-08-31

**Authors:** Jun-Nan Guo, Tian-Yi Xia, Shen-Hui Deng, Wei-Nan Xue, Bin-Bin Cui, Yan-Long Liu

**Affiliations:** ^1^ Department of Colorectal Surgery, Harbin Medical University Cancer Hospital, Harbin, China; ^2^ Department of Anesthesiology, The Fourth Affiliated Hospital of Harbin Medical University, Harbin, China

**Keywords:** colon adenocarcinoma, left-sided, right-sided, genomic instability, immunity, prognosis, therapeutic sensitivity

## Abstract

**Background:** The purpose of our study was to develop a prognostic risk model based on differential genomic instability-associated (DGIA) long non-coding RNAs (lncRNAs) of left-sided and right-sided colon cancers (LCCs and RCCs); therefore, the prognostic key lncRNAs could be identified.

**Methods:** We adopted two independent gene datasets, corresponding somatic mutation and clinical information from The Cancer Genome Atlas (TCGA) and Gene Expression Omnibus (GEO) databases. Identification of differential DGIA lncRNAs from LCCs and RCCs was conducted with the appliance of “Limma” analysis. Then, we screened out key lncRNAs based on univariate and multivariate Cox proportional hazard regression analysis. Meanwhile, DGIA lncRNAs related prognostic model (DRPM) was established. We employed the DRPM in the model group and internal verification group from TCGA for the purpose of risk grouping and accuracy verification of DRPM. We also verified the accuracy of key lncRNAs with GEO data. Finally, the differences of immune infiltration, functional pathways, and therapeutic sensitivities were analyzed within different risk groups.

**Results:** A total of 123 DGIA lncRNAs were screened out by differential expression analysis. We obtained six DGIA lncRNAs by the construction of DRPM, including AC004009.1, AP003555.2, BOLA3-AS1, NKILA, LINC00543, and UCA1. After the risk grouping by these DGIA lncRNAs, we found the prognosis of the high-risk group (HRG) was significantly worse than that in the low-risk group (LRG) (all *p* < 0.05). In all TCGA samples and model group, the expression of CD8^+^ T cells in HRG was lower than that in LRG (all *p* < 0.05). The functional analysis indicated that there was significant upregulation with regard to pathways related to both genetic instability and immunity in LRG, including cytosolic DNA sensing pathway, response to double-strand RNA, RIG-Ⅰ like receptor signaling pathway, and Toll-like receptor signaling pathway. Finally, we analyzed the difference and significance of key DGIA lncRNAs and risk groups in multiple therapeutic sensitivities.

**Conclusion:** Through the analysis of the DGIA lncRNAs between LCCs and RCCs, we identified six key DGIA lncRNAs. They can not only predict the prognostic risk of patients but also serve as biomarkers for evaluating the differences of genetic instability, immune infiltration, and therapeutic sensitivity.

## Introduction

Colon cancer (CC) is one of the most common cancers diagnosed in humans and, globally, there are more than 1.8 million new cases of this disease each year (Siegel et al., 2020; Verkuijl et al., 2021). Lately, immunotherapy has achieved breakthroughs and is considered a leading therapy against tumors. However, some CC patients show a low response and drug resistance (Wu, 2018). Traditional treatments such as surgery, radiotherapy, and chemotherapy are used to suppress cancers, but their long-term effects are difficult to predict. The differences of these phenomena are more obvious in the left-sided and right-sided CCs (LCCs and RCCs, respectively) (Grass et al., 2019). As mentioned in the literature review, LCC patients benefit more from chemotherapies and targeted therapies and have a better prognosis. RCC patients do not respond well to conventional chemotherapies but demonstrate more promising results with immunotherapies (Baran et al., 2018). It is well known that the differences in terms of molecular and clinical heterogeneity between LCCs and RCCs are complex, as are their occurrence, development, and response to treatment and prognosis (Blakely et al., 2020). Patients with RCC were found to have different molecular biological tumor patterns and a poorer prognosis than patients with LCC (Hansen and Jess, 2012). Thus, it is important to understand the underlying potential molecular mechanisms as they will influence the choice of treatments.

Recently, researchers have revealed that LCCs and RCCs are different in clinical and genomic characteristics (Mjelle et al., 2019). In addition to the microsatellite instability status, the identified differences include APC, TP53, RAS, and BRAF mutations (Guinney et al., 2015; Molina-Cerrillo et al., 2020; Requena and Garcia-Buitrago, 2020). The dissimilarities of gene expression patterns could be used to analyze LCCs and RCCs. It is mainly beneficial to doctors by selecting the most effective individualized treatment via the degree and nature of these molecular mutations. Therefore, while we are looking for novel and precise prognostic biomarkers, the utility is more vital for guiding targeted therapy.

Prior to cell division, the fidelity of our genome copies is remarkable in its consistency over time (Bebenek, 2008). However, these high fidelity processes can be compromised by a variety of genomic alterations that subsequently result in the development of cancer (Mardis, 2019). In CC, mutations in mismatch repair genes lead to functional defects, which can cause microsatellite instability (MSI). The distinction in MSI status is also one of the aspects that help to differentiate between LCCs and RCCs (Lichtenstern et al., 2020). In addition, a variety of biological processes are related to genome instability, such as abnormal transcription and post-transcriptional regulation and DNA damage regulation (Boulianne and Feldhahn, 2018). The latest findings disclosed that variations in the instability of genomes produce new antigens, which affect the immunophenotype and immunotherapy response (Mardis, 2019). Long non-coding RNAs (lncRNAs) are incapable of encoding proteins but they play an indispensable regulatory role in tumors. Currently, lncRNAs have been shown to be related to genome stability (Thapar, 2018). However, in LCCs and RCCs, the influence of differential genomic instability-associated (DGIA) lncRNAs on tumor-associated immune microenvironment has not been explored yet.

Therefore, in this research, we proposed to create a prognostic model and risk factor clustering containing key lncRNAs based on differentially expressed genes and genomic instability in the LCCs and RCCs in The Cancer Genome Atlas (TCGA) database; the leading goal of this study was to analyze the differences in immune infiltration between high- and low-risk groups (HRG and LRG, respectively) and to verify using the Gene Expression Omnibus (GEO) database. Moreover, we aimed to screen out new prognostic biomarkers related to genetic instability in LCCs and RCCs and to provide a molecular basis for identifying therapeutic sensitivities.

## Materials and Methods

The flowchart of the whole study is presented in Supplementary figure S1.

### Data Collection

In this research, we selected two independent gene datasets from different high-throughput platforms, including 473 colon adenocarcinoma (COAD) samples from TCGA (https://portal.gdc.cancer.gov/) and 156 COAD samples from GEO (http://www.ncbi.nlm.nih.gov/geo/) (GSE103479). The downloaded data included paired lncRNA and mRNA expression profiles, somatic mutation information, and clinical information. The RCC tumors arise from ascending colon, and proximal two-thirds of the transverse colon and the LCC tumors arise from the descending and sigmoid colon and distal one-third of the transverse colon (Baran et al., 2018). After screening based on CCs location, there were a total of 411 samples with integrated information available for analysis, of which 322 were from TCGA and 89 from GEO. The TCGA samples were divided into two groups randomly—model group and internal verification group. To ensure the undifferentiated clustering, we performed an analysis to determine the differences in stratification of various clinical factors. The GEO sample was used as the external validation group to verify the accuracy of prognostic lncRNAs. The analysis excluded RNA that was undetectable in more than 10% of the samples. Concerning each dataset, the gene ID was converted to the corresponding gene symbol according to the corresponding annotation package.

### Identification of Differential Genomic Instability-Associated lncRNAs From LCCs and RCCs

Initially, we examined differentially expressed genes (DEGs) in LCCs and RCCs from TCGA by the R package “Limma” (|log2foldchange| >0.5, false discovery rate (FDR) <0.05) (Ritchie et al., 2015). These DEGs were distributed by the human genome annotation package into mRNAs and lncRNAs. In order to assess the genomic instability, we proposed a mutator hypothesis-derived calculation method: we determined the cumulative number of somatic mutations (CNSMs) on the basis of the number of changed sites in each gene of each sample and categorized the patients in descending order. The top 25% of patients were titled with genomic unstable like (GU) group and the last 25% as genomic stable like (GS) group. The differentially expressed lncRNAs of the two groups were evaluated and named as DGIA lncRNAs from LCCs and RCCs (| log2foldchange | >0.5, FDR <0.05).

### Cluster and Analyze the TCGA Samples According to DGIA

Hierarchical cluster test (HCA) was performed to verify the grouping effect of DGIA IncRNAs and to batch all TCGA samples according to DGIA by the R package “sparcl” (Witten and Tibshirani, 2010). HCA is an approach commonly used to classify similar samples or variables using Euclidean distances and Ward’s linkage method. The samples were classified into GU group and GS group by clustering. Subsequently, we explored the two groups on the CNSMs by univariate analysis.

### Functional Enrichment Analysis

To investigate the potential functions of DGIA lncRNAs, two methods were applied to identify mRNAs that were more likely to be co-expressed with them. The first one was used to analyze the co-expressing relationship between lncRNAs and mRNAs by Pearson’s correlation tests. Here, we designated that the top 10 mRNAs with the highest coefficients have a strong co-expressing relationship with each DGIA lncRNA.

The second one was used to analyze the DEGs between LCCs and RCCs through weighted gene co-expression network assessment by the R package “WGCNA” (Langfelder and Horvath, 2008). At first, the construction of an adjacency matrix (AM) of genes was done using the power function with an appropriate power index selected. Then, AM was converted into a topological overlap matrix. Finally, the gene consensus modules were collected and the correlation analysis was performed with CNSMs. The mRNAs in the modules with the highest absolute correlation coefficient with CNSMs were selected for further examination.

Overall, the intersection of the mRNAs was screened by two methods and we employed Gene Ontology (GO) functional annotations and Kyoto Encyclopedia of Genes and Genomes (KEGG) pathway enrichment analysis by R package “clusterProfiler” (Yu et al., 2012).

### Construction of DGIA lncRNAs Related Prognostic Model

To check the effect of DGIA lncRNAs on the prognosis, DGIA lncRNAs were secluded by univariate Cox proportional hazard regression analysis (COX). LncRNAs with *p* < 0.05 in univariate COX were retained and multivariate COX was performed in the model group by the R package “glmnet” (Friedman et al., 2010). The risk scores (RS) of the model group and the internal validation group were estimated according to the coefficient of each lncRNAs within the model. The patients in TCGA were separated into HRG and LRG with poor prognosis.

### Validation of the DGIA lncRNAs in DRPM

Log-rank test was used to disclose the difference in survival of HRG and LRG in the model group and internal validation group by R packages “survcomp” (Schröder et al., 2011). Simultaneously, the predictive effect of DRPM was figured out through the receiver operating characteristics curve (ROC) and the area under the ROC curve (AUC) by the R package “survivalROC” (Heagerty and Zheng, 2005). Additionally, univariate and multivariate COX was utilized to verify the independent predictive effect of the RS obtained by the model.

In the external verification group, DGIA lncRNAs were employed in DRPM to construct a prognostic model again by multivariate COX. The Log-rank test was also used for survival analysis, and time-dependent ROC (timeROC) of 1, 3, and 5 years was plotted. The purpose was to verify the accuracy of prognostic DGIA lncRNAs.

The survival curves of DGIA lncRNAs in DRPM were plotted and the differences were analyzed by log-rank test. The R package “maxstat” (Laska et al., 2012) was performed to obtain the best cut-off value.

### Immune Infiltration and Gene Set Enrichment Analysis in HRG and LRG

The R package “CIBERSORT” (Newman et al., 2015) was employed in the TCGA samples to estimate the relative infiltration abundance of 22 immune cells and to assess the variations in the various immune cells’ infiltration of HRG and LRG. The results with *p* < 0.05 were retained. “CIBERSORT” calculated the *p* value of the deconvolution for each sample by Monte Carlo simulation to provide the assessed confidence. The differences in the abundance of 22 immune cells in HRG and LRG were examined by the Wilcoxon rank-sum test.

Besides, to study the differences in biological functions of genes between HRG and LRG, we downloaded the biological process (BP), molecular function (MF) datasets related to GO, and the KEGG dataset. GSEA was performed using the Bioconductor package “fgsea” (Subramanian et al., 2005) with 10,000 permutations between LRG and HRG. The threshold values were *p* < 0.05.

### Related Analysis of Therapeutic Sensitivity

To evaluate the application of the DRPM and DGIA lncRNAs in clinical therapy of CC, we analyzed the therapeutic sensitivity from three aspects: chemotherapy, targeted inhibitors (TIs) therapy, and immunotherapy. Gene expression data and chemotherapeutic drug response data were downloaded from CellMiner™ (https://discover.nci.nih.gov/cellminer/); these data were from the same batch. We deleted drugs without FDA approval or clinical trials and selected chemotherapy drugs for CC. Then, we extracted the lncRNAs and co-expressed mRNA from the gene expression data and analyzed the correlation between their expression and drug sensitivity.

In addition, we calculated the concentration causing 50% reduction growth (IC50) of TIs by R package “pRRophetic” (Geeleher et al., 2014), including vascular endothelial growth factor receptor (VEGFR), Hedgehog (HH), and Wnt inhibitors. Wilcoxon rank-sum test was used to compare the IC50 difference between HRG and LRG. Finally, we analyzed the differences in gene expression of the six immunosuppressive checkpoints in HRG and LRG.

## Result

### DEGs and DGIA lncRNAs in LCCs and RCCs

**FIGURE 1:**
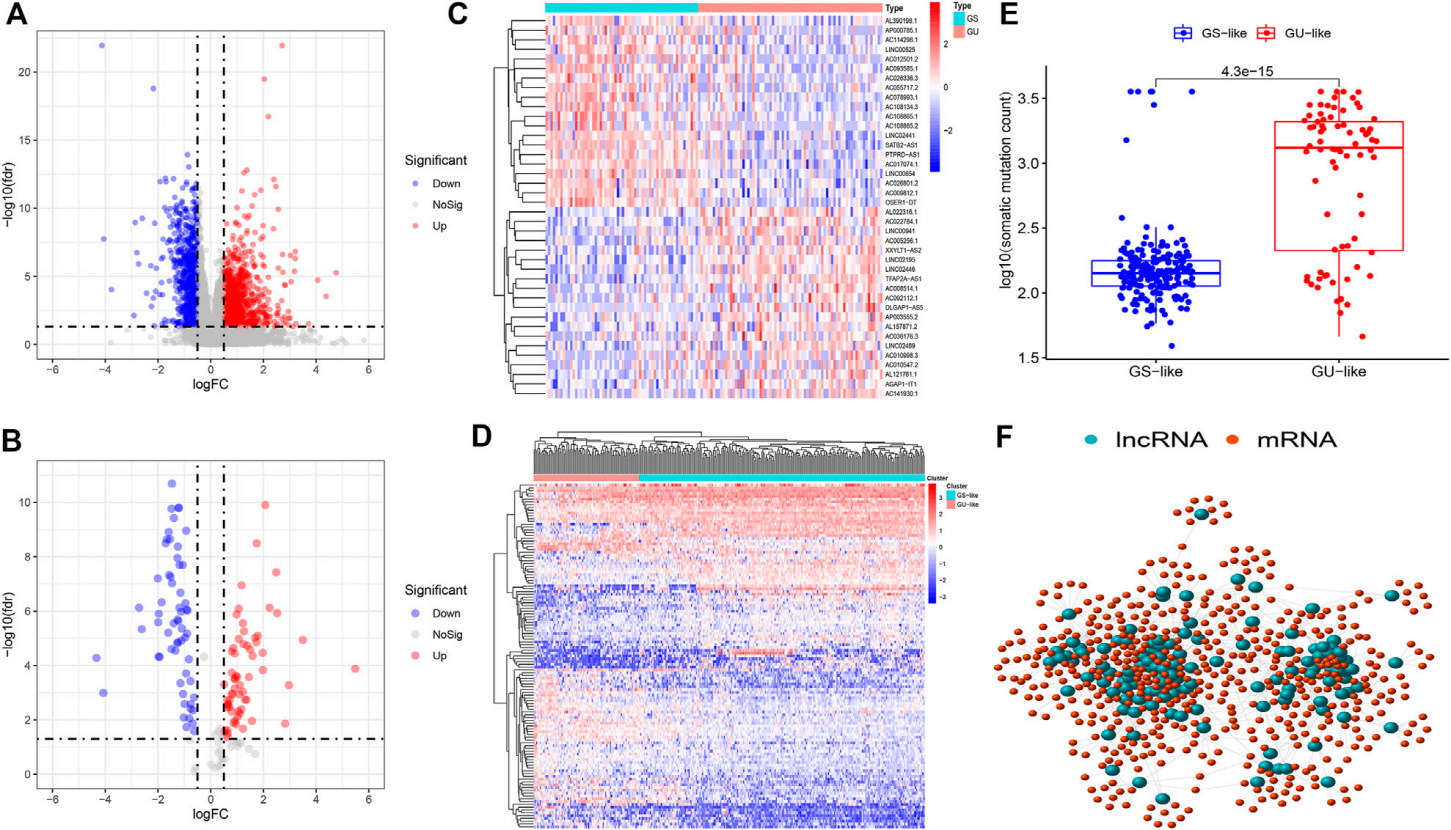
**(A)** Differentially expressed genes between LCCs and RCCs. **(B)** Differentially expressed DGIA lncRNAs between GU group and GS group. Red and blue circles indicate high and low genes expression, respectively. **(C)** Heat map depicts the differentially expressed DGIA lncRNAs in TCGA patients. **(D)** Unsupervised clustering of TCGA patients based on the expression pattern of 128 candidate DGIA lncRNAs. **(E)** In boxplot, cumulative number of somatic mutations in the GU-like group is significantly higher than that in the GS-like group **(F)** Co-expression network of DGIA lncRNAs and intersection of mRNAs based on two methods. The red circles represent lncRNAs, and the blue circles represent mRNAs. LCCs and RCCs: left- and right-sided colon adenocarcinoma; DGIA lncRNAs: genomic instability-associated long non-coding RNAs; GU: genomic unstable; GS: genomic stable.

Firstly, we selected, separated, and bundled TCGA samples in furtherance of segregating DGIA IncRNAs from DEGs of LCCs and RCCs. Soon after, the corresponding gene expression data were standardized and analyzed. We obtained 1724 DEGs (Figure 1A), including 1,325 mRNAs and 399 lncRNAs. According to the CNSMs, the top 25% (n = 75) and last 25% (n = 62) patients were labeled as GU group and GS group, respectively. By comparing the lncRNAs between these two groups, 123 DGIA lncRNAs were attained, of which 63 lncRNAs showed upregulation whereas 60 exhibited downregulation in the GU group (Figures 1B,C). Based on these DGIA lncRNAs, we carried out unsupervised HCA on TCGA specimens and distributed them into the GU group and GS group (Figure 1D). The CNSMs in both groups were significantly different with the median higher in the GU group compared to the GS group (Figure 1E). These findings conclusively depicted that the selected DGIA lncRNAs had a valid classification effect.

### Functional Enrichment Analysis for DGIA lncRNAs

To explore the functions and pathways concerned with 123 DGIA lncRNAs, we operated functional enrichment analysis on protein-coding genes (PCGs) co-expressed with DGIA lncRNAs. The first method’s procedure included a correlation analysis between the selected DGIA lncRNAs and 1,325 differential mRNAs from LCCs and RCCs. The PCGs of DGIA lncRNAs, that is, the top 10 mRNAs with the strongest correlation with each lncRNAs, were achieved.

**FIGURE 2:**
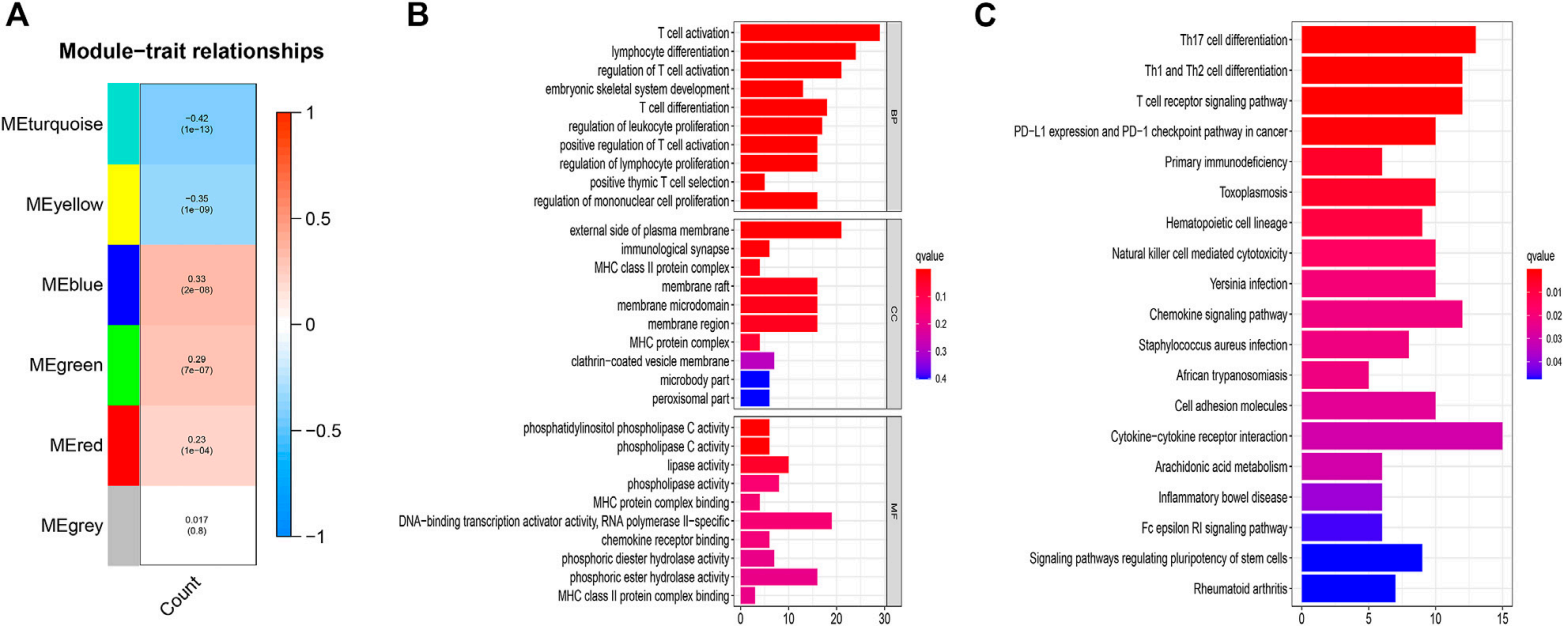
**(A)** Correlation between the gene modules and CNSMs. Each cell contains a corresponding correlation coefficient and *p* value. The correlation coefficient decreased in size from red to blue. **(B)** GO functional annotations for mRNAs co-expressed with lncRNAs. **(C)** KEGG enrichment analysis for mRNAs co-expressed with lncRNAs. CNSMs: cumulative number of somatic mutations; GO: Gene Ontology; KEGG: Kyoto Encyclopedia of Genes and Genomes.

The second method disclosed, after constructing the co-expression network (Supplementary Figure S2), the blue module with the highest positive correlation, and the turquoise module with the highest negative correlation (Figure 2A). We intersected the selective mRNAs from the blue and turquoise modules with PCGs chosen by the first method. Thereby, these genes were used to construct a lncRNAs-mRNA co-expression network (Figure 1F).

The results of functional enrichment analysis with the intersection PCGs comprised DNA-binding transcription activator activity, RNA polymerase II-specific, and various phospholipase-related enzyme activities. These molecular functions are closely associated with the formation and development of genomic instability. More importantly, the enrichments of biological processes are mainly related to immune processes, such as T cell activation, lymphocyte differentiation, regulation of T cell activation. (Figure 2B). KEGG enrichment analysis displayed that regulatory pluripotency of both stem cells signaling pathways and immune-related pathways, including Th17 cell differentiation, Th1 and Th2 cell differentiation, PD−L1 expression, and PD−1 checkpoint pathway in cancer, were significantly enriched (Figure 2C). These results indicated that 123 DGIA lncRNAs not only cause genomic instability but also influence the regulation of the immune system. The variation in the expression of these 123 DGIA IncRNAs potentially disturbs the balance of co-expressed PCGs regulatory network and, consequently, causes instability in the cell genome. It also affects the killing of tumors by immune cells, mostly by proliferation, differentiation, activation, and receptor recognition of T cells. Thus, these DGIA lncRNAs play an essential role in immune regulation while affecting gene instability.

### Construction of DRPM Using DGIA lncRNAs

The samples from TCGA were randomly and uniformly arranged into model group (n = 162) and validation group (n = 160). The clinical factors were not statistically significantly different between each group (all *p* > 0.05) (Supplementary Table S1). In the model group, we employed univariate and multivariate COX to assort and construct DRPM with 123 DGIA lncRNAs, and six prognostic-related DGIA lncRNAs with corresponding risk coefficients were determined (Table 1). All patients in TCGA were divided into HRG and LRG on the basis of the median of RS (0.851) measured by DRPM in the model group (Supplementary Table S2).

**TABLE 1:** DRPM information including six DGIA lncRNAs.

LncRNAs	Coefficients	HR	95% CI lower	95% CI upper	*p* value
NKILA	0.198	1.219	1.027	1.447	0.024
AC004009.1	0.316	1.371	1.128	1.668	0.002
AP003555.2	0.377	1.457	1.227	1.731	<0.001
BOLA3-AS1	0.329	1.390	1.038	1.860	0.027
LINC00543	0.074	1.077	1.007	1.152	0.030
UCA1	0.013	1.014	1.004	1.023	0.004

CI: confidence interval; DGIA: differential genomic instability-associated; DRPM: DGIA lncRNAs related prognostic model; HR: hazard ratio; LncRNAs: long non-coding RNAs.

### Validation of the DRPM

**FIGURE 3:**
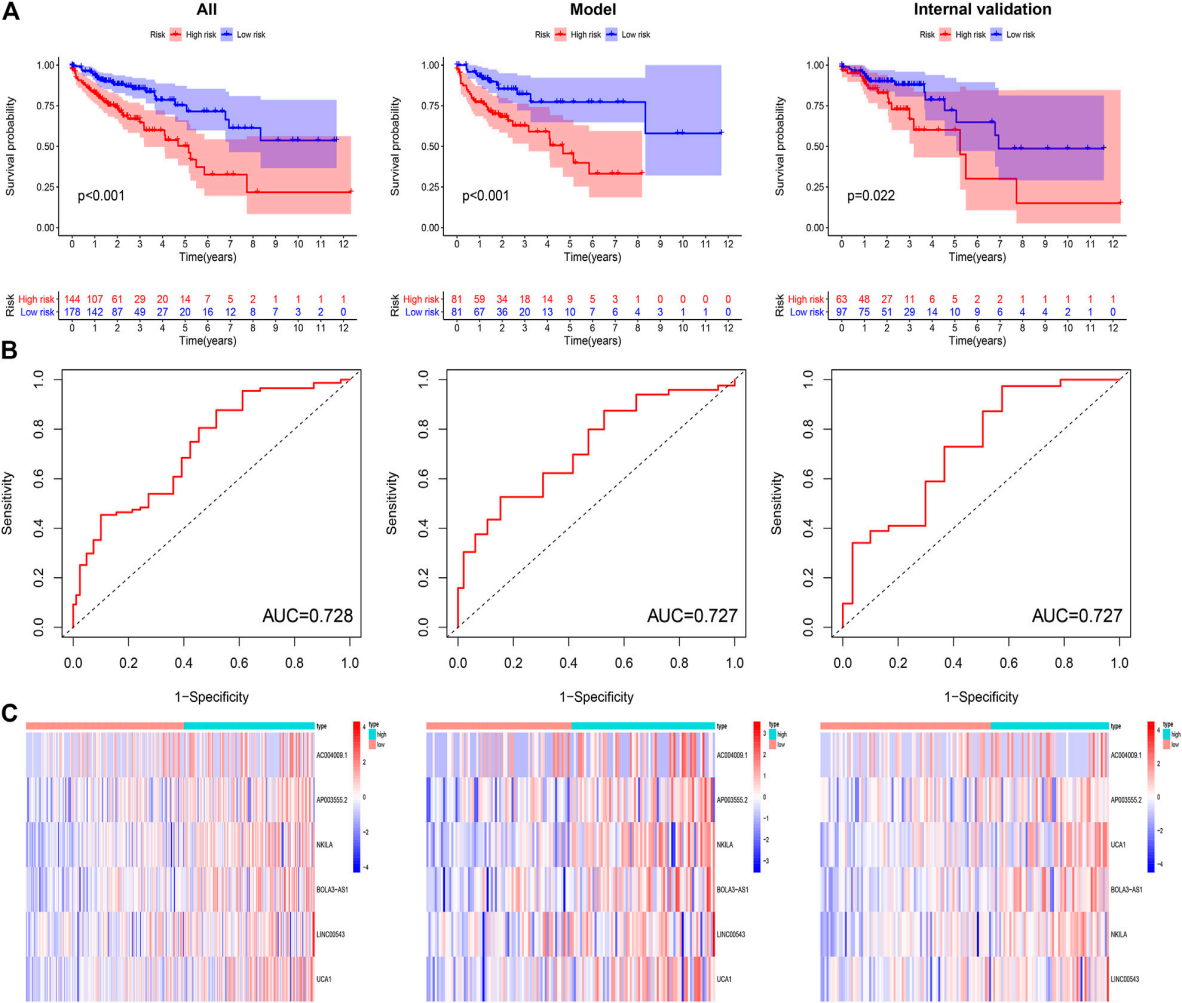
**(A)** Kaplan–Meier curves of overall survival in different groups. **(B)** ROC curves in different groups. **(C)** The heat map of six key lncRNA expression patterns with increasing risk score. ROC: receiver operating characteristics.

To confirm the anticipated effects of DRPM, we conducted the Kaplan–Meier test to plot a survival curve. The results demonstrated that the survival outcomes of HRG were worse than those in LRG (all *p*<0.05) (Figure 3A). The ROC curves plotted for patients in different groups confirmed the consistency and categorization effects of DRPM. The AUC were shown in the figures respectively (Figure 3B). Using RS, we organized the patients into different groups and detected changes in the expression level of the prognostic DGIA lncRNAs. The heat map presented the increment in the expression levels of six lncRNAs in HRG (Figure 3C).

To verify the independent predictive effects of RS, we combined RS with clinical factors for univariate and multivariate COX analysis. These clinical factors were age, gender, and TNM stage. The results indicated that RS was an independent prognostic factor (Table 2). Besides, to assess the risk clustering ability of DRPM in different strata, we separately stratified age (<65 years and ≥65 years), gender (male and female), and clinical stage (stages I-II and stages III-IV). The survival curves of HRG and LRG were plotted through the stratification of different clinical factors. HRG and LRG exhibited a significant difference in overall the strata of age, gender, and stages I-II (all *p* < 0.05) (Supplementary Figure S3). In the strata of stages III-IV, the difference was very close but was not statistically significant (*p* = 0.077) **(** Supplementary Figure S3). In summary, the DRPM revealed a consistent and promising prognostic evaluation ability in different strata.

**TABLE 2:** Univariate and multivariate COX of prognostic factors in different groups.

Factors	Univariate COX				Multivariate COX			
	HR	95% CI lower	95% CI upper	*p* value	HR	95% CI lower	95% CI upper	*p* value
All patients in TCGA (n = 322)								
Age	1.031	1.010	1.053	0.004	1.043	1.021	1.066	<0.001
Gender	1.330	0.828	2.134	0.238	1.149	0.698	1.891	0.584
T	3.555	2.155	5.864	<0.001	2.897	1.601	5.240	<0.001
N	1.898	1.438	2.506	<0.001	1.132	0.804	1.595	0.477
M	4.484	2.740	7.337	<0.001	2.985	1.603	5.559	0.001
Risk score	1.228	1.161	1.300	<0.001	1.196	1.127	1.269	<0.001
Model group (n = 162)								
Age	1.024	0.996	1.053	0.094	1.033	1.002	1.065	0.038
Gender	0.734	0.393	1.369	0.331	0.758	0.388	1.480	0.417
T	2.359	1.235	4.507	0.009	1.465	0.702	3.060	0.309
N	1.904	1.315	2.756	0.001	1.216	0.763	1.940	0.411
M	4.654	2.451	8.838	<0.001	3.405	1.500	7.726	0.003
Risk score	1.235	1.158	1.317	<0.001	1.160	1.078	1.249	<0.001
Internal validation group (n = 160)								
Age	1.040	1.006	1.076	0.021	1.063	1.026	1.102	0.001
Gender	3.060	1.361	6.878	0.007	2.179	0.904	5.248	0.083
T	6.649	3.078	14.362	<0.001	9.612	3.662	25.230	<0.001
N	2.074	1.313	3.276	0.002	1.378	0.744	2.555	0.308
M	4.349	1.992	9.493	<0.001	2.299	0.767	6.891	0.137
Risk score	1.178	1.033	1.343	0.015	1.250	1.088	1.436	0.002

COX: Cox proportional hazard regression analysis.

### Validation of the Prognostic DGIA lncRNAs

**FIGURE 4:**
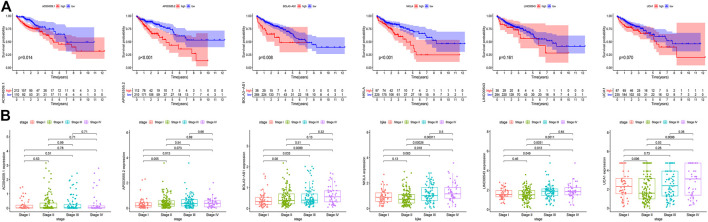
**(A)** Kaplan–Meier curves of overall survival in six key DGIA lncRNAs. **(B)** The correlation between six key DGIA lncRNAs and pathologic stages.

To verify the accuracy of prognostic DGIA lncRNAs, we plotted survival curves for the lncRNAs in TCGA samples. In AC004009.1, AP003555.2, BOLA3-AS1, NKILA, LINC00543, and UCA1, the prognosis of the high expression group was worse compared to the low expression group (all *p* < 0.05) (Figure 4A). Also, we investigated the correlation between these lncRNAs at different stages, and the results indicated that the expression levels of AP003555.2, BOLA3-AS1, NKILA, LINC00543, and UCA1 were significantly different between the least two stages (Figure 4B).

**FIGURE 5:**
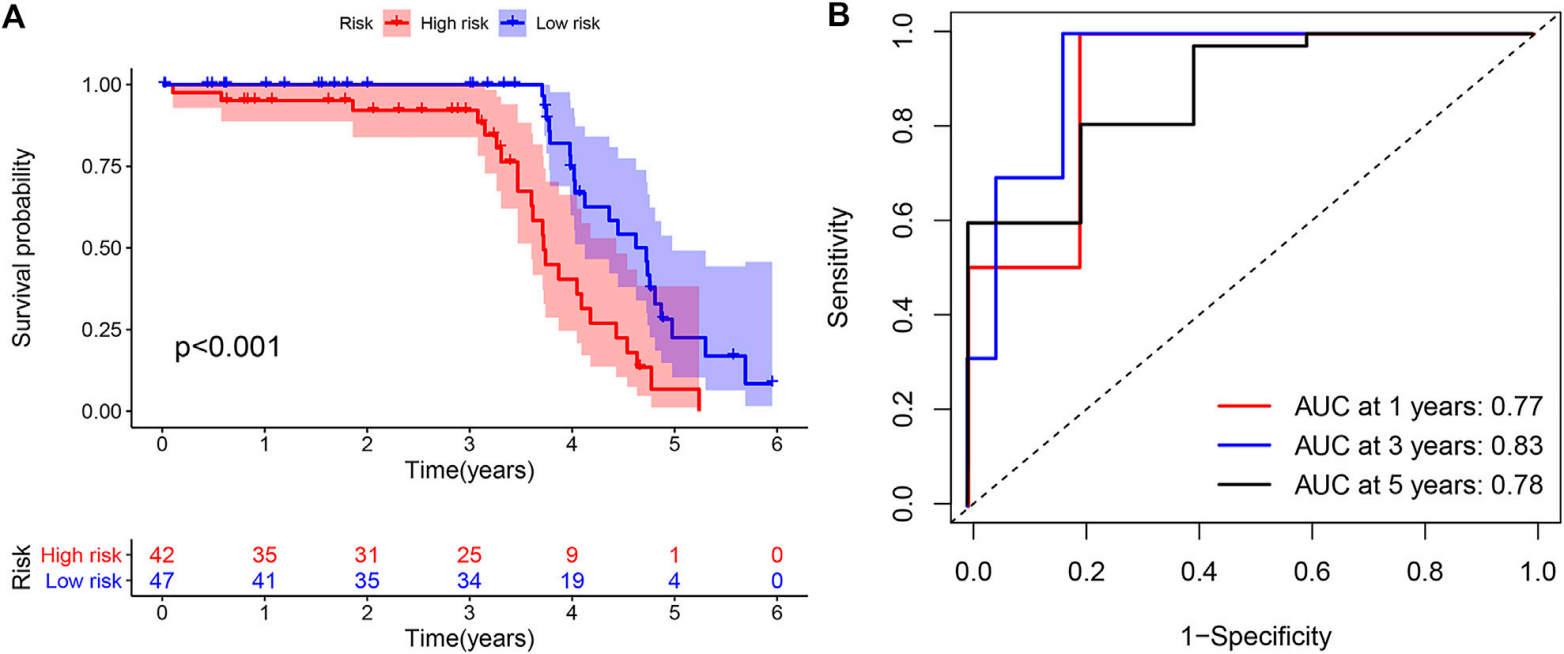
**(A)** Kaplan–Meier curve of overall survival in external validation group. **(B)** TimeROC curves for 1, 3, 5 years in external validation group. TimeROC: time-dependent ROC.

Meanwhile, in the external validation group from GEO, we constructed a model and grouped patients with four prognostic DGIA lncRNAs, including BOLA3-AS1, NKILA, LINC00543, and UCA1. The prognosis of HRG was also worse than that of LRG (*p* < 0.001) (Figure 5A). The timeROC of 1, 3, and 5 years proved that the model had a promising classification effect and that of 3 years displayed an optimum effect (AUC = 0.83) (Figure 5B).

### Immune Infiltration and GSEA Within Different Risk Groups

**FIGURE 6:**
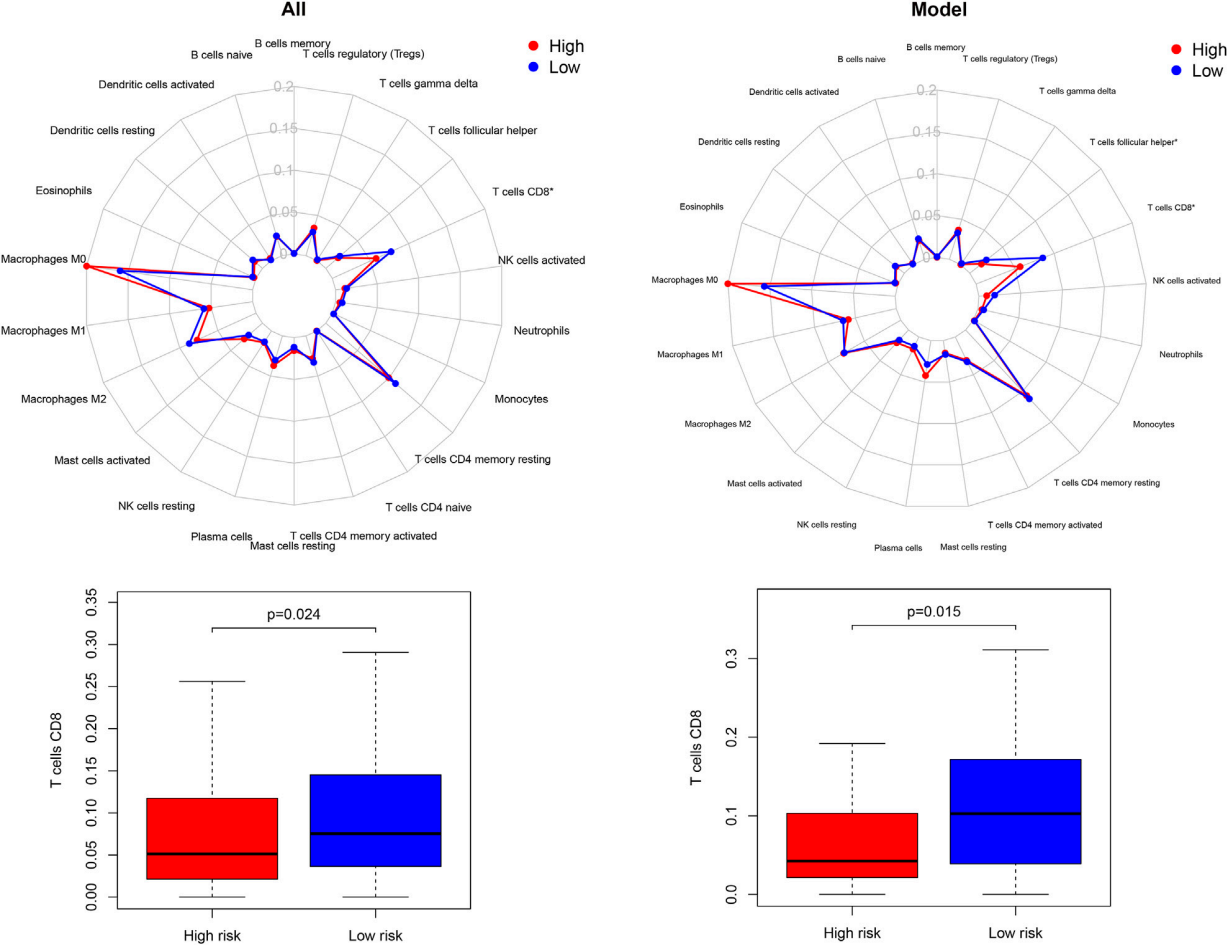
The differences of 22 immune cell types abundance within different risk groups.

The enrichment investigation mentioned above demonstrated that DGIA lncRNAs also influence immune regulation. Hence, we evaluated the differences in the infiltration of 22 immune cells in HRG and LRG according to the results of CIBERSORT. The expression of CD8^+^ T cells in all TCGA samples and model group was lower in HRG than in LRG (Figure 6). CD8^+^ T cells are cytotoxic immune cells that can kill tumor cells directly, and their abundance difference indicated the immune-related cause of the prognostic difference in HRG and LRG.

**FIGURE 7:**
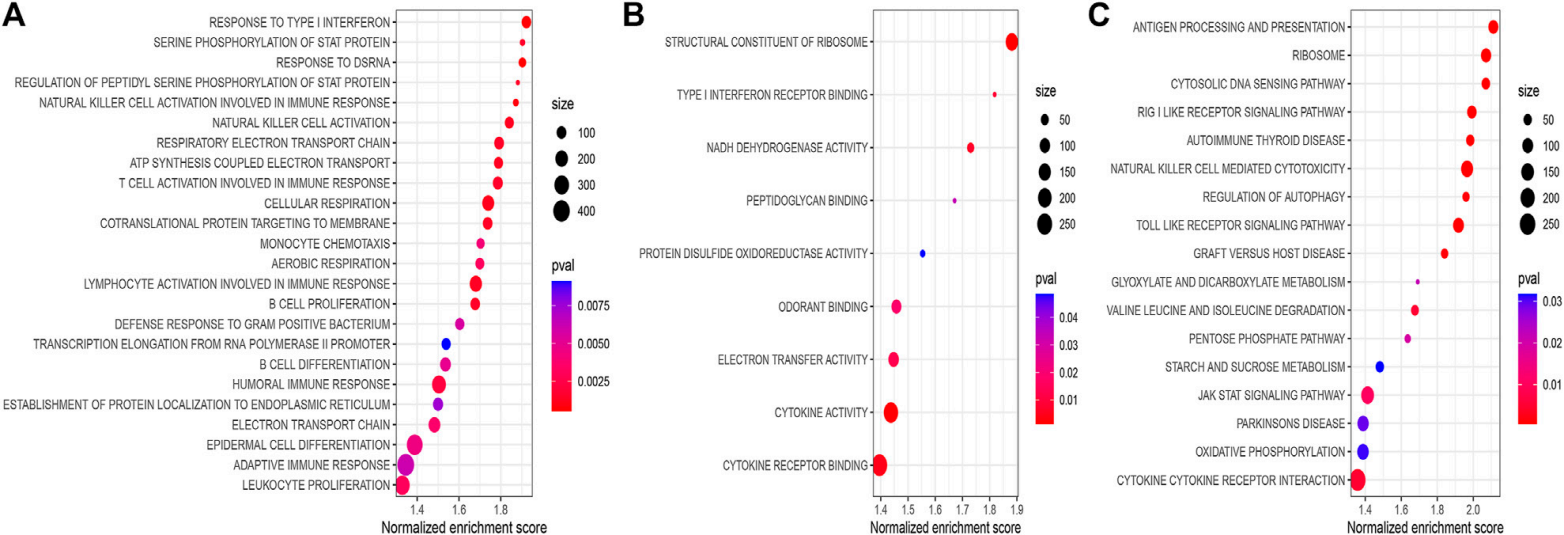
**(A)** BP of GO-related GSEA between different risk groups. **(B)** MF of GO-related GSEA between different risk groups. **(C)** KEGG-related GSEA between different risk groups. BP: biological process; MF: molecular function.

To explore the significantly altered MF, BP, and pathways in HRG and LRG, we performed GO- and KEGG-related GSEA. Mainly, immune and genomic instability-related pathways in LRG were significantly enriched. In GO enrichment terms, immune-related pathways encompassed response to type I interferon (IFN-Ⅰ), natural killer cell activation, and T cell activation involved in the immune response. Simultaneously, some genomic instability-related pathways were also significantly enriched, including structural constituent of ribosome, transcription elongation from RNA polymerase II promoter, response to double-strand RNA (dsRNA), and some energy-related pathways in glucose metabolism (Figures 7A,B). In KEGG enrichment terms, apart from the regulation of autophagy and cytosolic DNA sensing pathway involved with genomic instability, there were also immune-related pathways, including antigen processing and presentation, and enriched cytokine receptor interaction (Figure 7C). Finally, we noticed that the CNSMs of LRG were significantly higher compared with HRG (all *p* < 0.05) (Supplementary Figure S4).

### Sensitivity of Different Therapies Within Different Risk Groups

**FIGURE 8:**
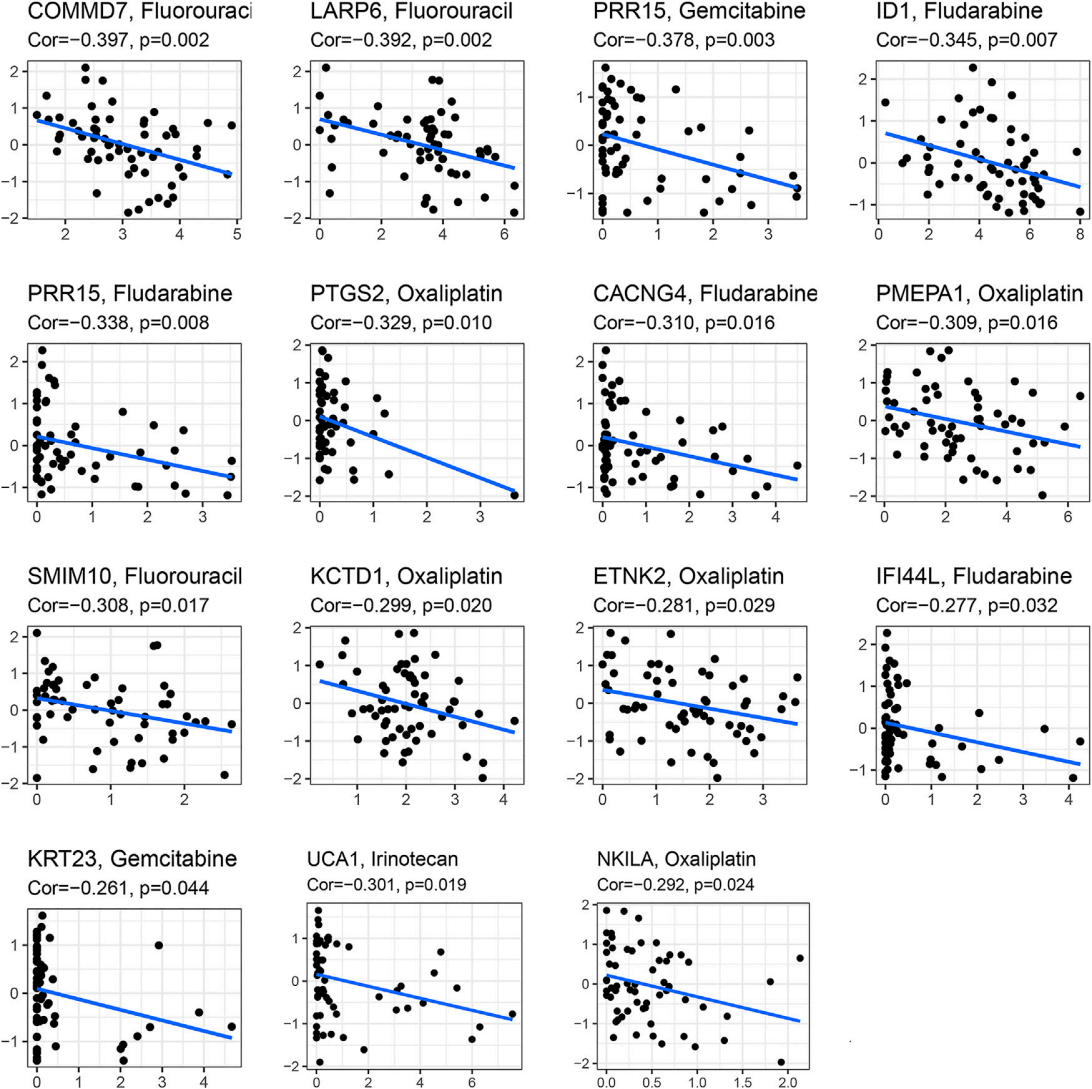
The correlation analysis in the expression of DGIA lncRNAs and co-expressed mRNA with a sensitivity of chemotherapy drugs.

**FIGURE 9:**
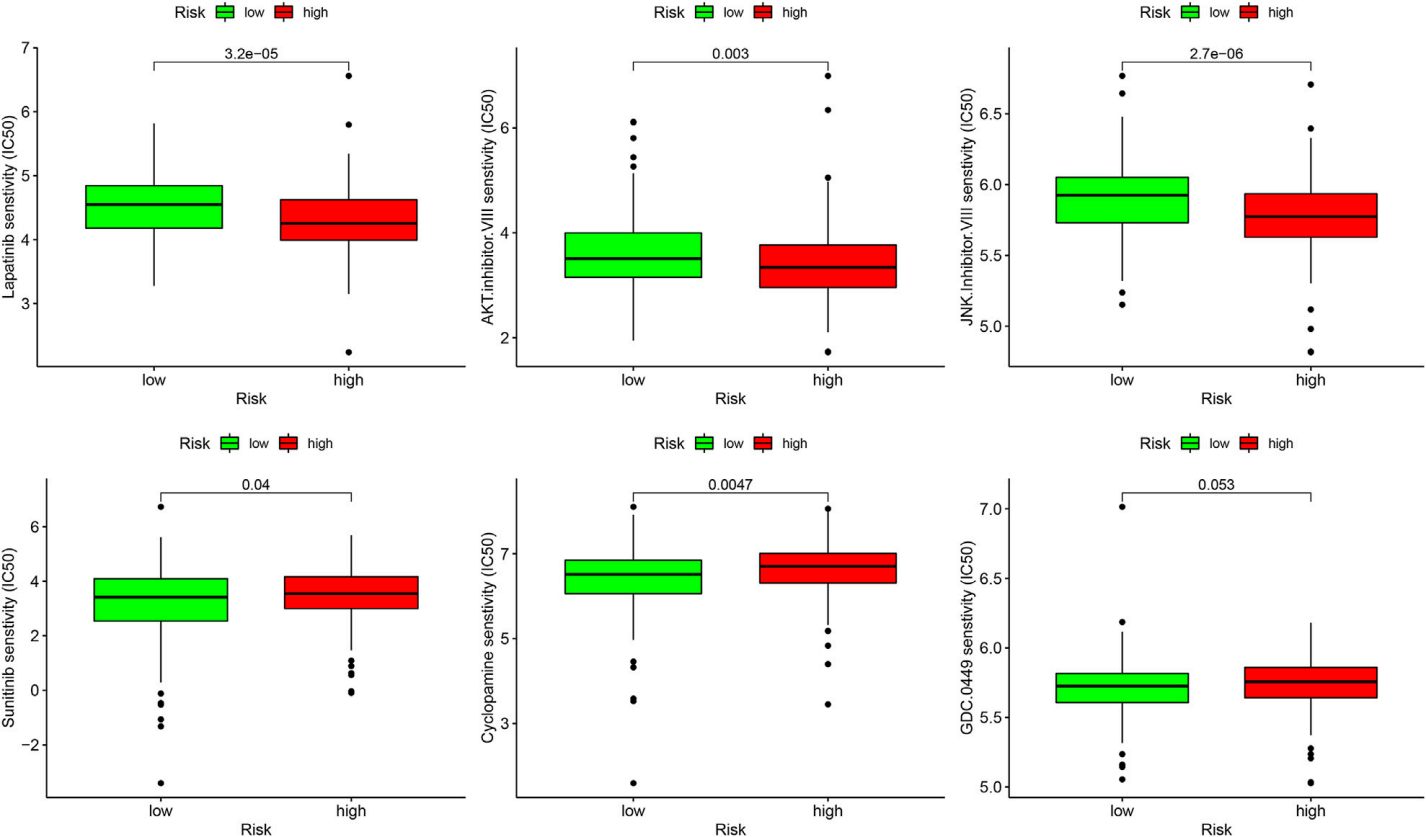
The sensitivity difference of multiple targeted inhibitors within different risk groups.

**FIGURE 10:**
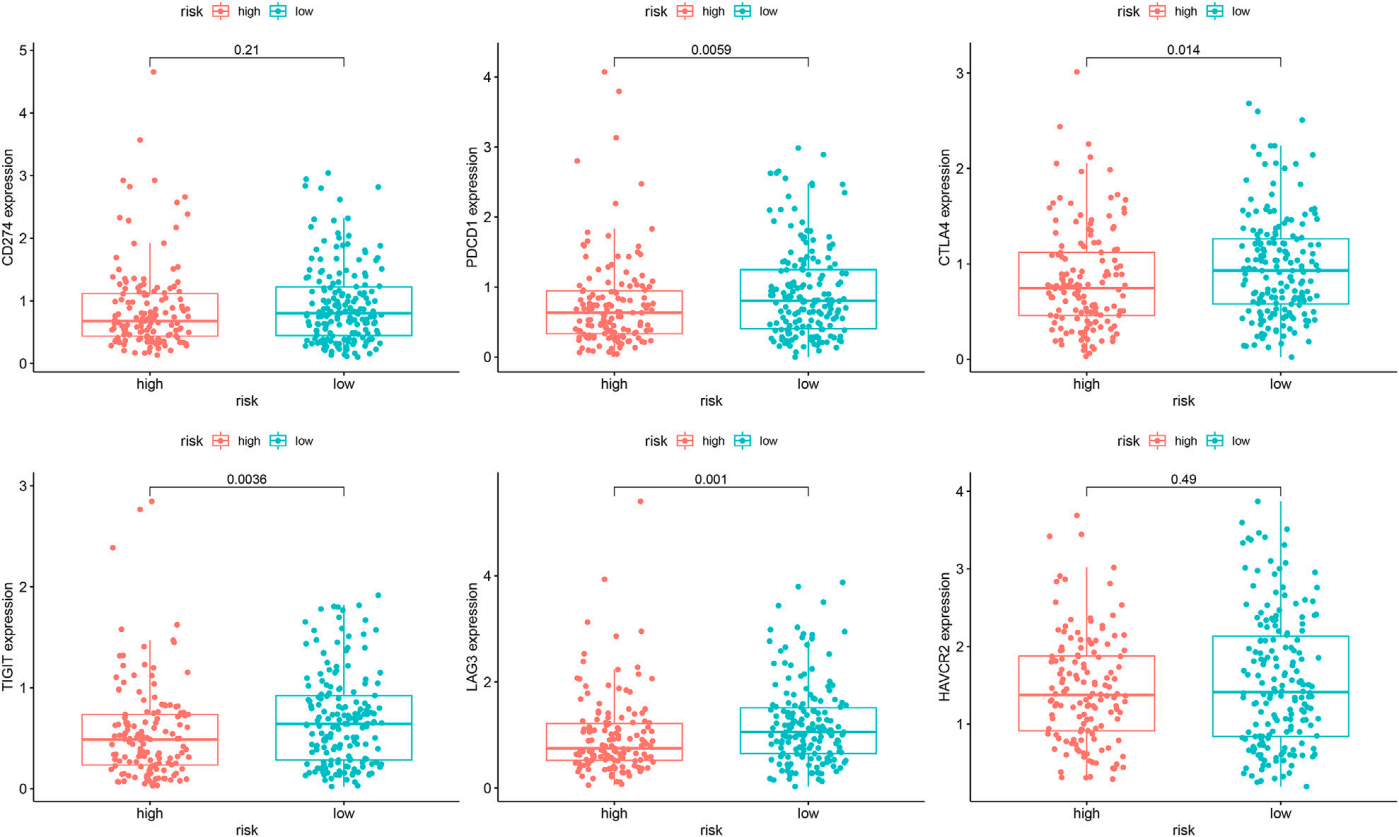
The difference of the expression of immunosuppressive checkpoints within different risk groups.

In the analysis of chemotherapy, we found that key DGIA lncRNAs and their co-expressed PCGs could reduce the sensitivity of most chemotherapy drugs, including oxaliplatin, fluorouracil, and irinotecan (new drug for CC) (Figure 8). As for TIs, different sensitivities were shown in HRG and LRG (Figure 9). In lapatinib (epidermal growth factor receptor inhibitor), AKT inhibitor VIII, and JNK Inhibitor VIII, the median IC50 of the LRG was significantly higher than that of HRG (all *p* < 0.05). In sunitinib (VEGFR inhibitor), cyclopamine (HH signaling inhibitor), and GDC.0449 (HH signaling inhibitor), the median IC50 of HRG was significantly higher than that of LRG. As for the immunosuppressive checkpoints, the expressions of PDCD1, CTLA4, TIGIT, and LAG3 were higher in LRG than those in HRG (Figure 10), which indicated that LRG patients could benefit potentially from immunotherapy. These results proved that the DRPM and key DGIA lncRNAs were useful in clinical therapy to some extent, and they provided predictive value for the sensitivity of multiple drugs.

## Discussion

The crucial role of the primary site in treatment decision-making has been progressively clarified. Around 2015, the “dispute between LCC and RCC” become one of the hotspots in CC. Tumor primary site is considered an independent prognostic factor for CC in stages III/IV. The prognosis of RCC is significantly worse than that of LCC, which is not related to treatment. Additionally, RCC acts as a negative predictor of EGFR-targeted therapy (Benson et al., 2017). As a matter of fact, LCC and RCC are inconsistent in many aspects (e.g., embryonic origin, anatomical blood supply, and clinical manifestations). However, the critical culprit could cause the difference in treatment response, and the prognosis is the molecular biological characteristics (Stintzing et al., 2017). Thus, drawing upon primary site alone is inadequate to formulate a treatment strategy and evaluate prognosis. Lately, it has been proclaimed that genomic instability is one of the key prognostic factors for most cancers (Andor et al., 2017). Various assays were used to assess the genomic instability by estimating the expression of certain characteristic proteins and gene mutations (Cortes-Ciriano et al., 2017; Davies et al., 2017). Moreover, with the development of gene sequencing technology, the detection of genomic instability has achieved an increased resolution (Stadler et al., 2016). In recent times, researchers have put a great effort to identify PCGs and microRNAs and to find biomarkers related to genomic instability and prognosis (Mettu et al., 2010; Habermann et al., 2011). Simultaneously, the increasing number of studies on lncRNAs also makes researchers aware of their role in genomic stability. Although many works have been done by scientists, identification of DGIA lncRNAs in LCCs and RCCs and their relationship in terms of immunity are still rarely mentioned. Therefore, we explored the influence of genomic instability in LCCs and RCCs, as well as the key prognostic DGIA lncRNAs.

We identified 123 DGIA lncRNAs from the DEGs of LCCs and RCCs. The functional analysis on their co-expressed PCGs surprisingly affirmed that these DGIA lncRNAs potentially influenced the genomic instability and immune functions through PCGs. In terms of molecular functions, the accumulation of errors during DNA transcription under the action of RNA polymerase is the source of genomic instability in all organisms (Khristich and Mirkin, 2020). Additionally, phospholipase C participates in numerous physiological processes within the cell, especially signal transduction pathways that regulate cell functions and proliferation (Jang et al., 2018; Owusu Obeng et al., 2020). These processes are also involved with genomic mutations and even lead to cancers (Koss et al., 2014). Other enriched pathways are mainly related to the positive activation and differentiation of T cells. Thus, we suspected that genomic instability could potentially cause differences in the prognosis and immunity of LCCs and RCCs.

Moreover, we investigated whether DGIA lncRNAs can identify differences in immunity while predicting clinical outcomes. Upon identification of DRPM containing six key lncRNAs, we successfully divided the patients into HRG and LRG with poor prognosis. From the study of differences in immune infiltration and the GSEA between HRG and LRG, it has been concluded that some pathways related to genetic instability in the LRG are significantly enriched, including regulation of autophagy and glucose metabolism-related pathways (Figure 7C). Genomic instability could induce the production of a large number of misfolded proteins, and autophagy may result in the degradation of ubiquitinated and misfolded proteins (Matsumoto et al., 2011). Autophagy has also been reported to be involved in regulating the number of centrosomes during cell division to maintain genomic instability (Watanabe et al., 2016). Besides, some pathways are associated with both genetic instability and immunity, such as the cytosolic DNA sensing pathway, response to dsRNA, RIG-Ⅰ like receptor signaling pathway, and Toll-like receptor signaling pathway (Figures 7A,C). In somatic cells, cytoplasmic DNA sensors, after identifying the double-strand DNA (dsDNA), activate the cytosolic DNA sensing pathway and innate immune responses (Kwon and Bakhoum, 2020). These dsDNA may be endogenous and are the yields of inaccurate replication of mitochondrial DNA (mtDNA) or micronuclear DNA (Kawai and Akira, 2010; Dhir et al., 2018). dsDNA can be transformed into dsRNA by the action of RNA polymerase III for recognition by the RNA sensor RIG-I (Hur, 2019; Samuel, 2019). dsRNA can also be recognized by Toll-like receptors to induce inflammatory cytokines and IFN-Ⅰ (Kawai and Akira, 2010; Miyake et al., 2018). Some pieces of research disclosed that dsRNA accumulates in the mitochondria as a result of gene deletion during transcription of mtDNA, which promotes the production of IFN-Ⅰ (eliciting innate immune response) after being recognized (Dhir et al., 2018). The evidence and our enrichment results explain the potential mechanism of immune activation by genomic instability.

The immune-related pathways are also significantly enriched and CD8^+^ T cell infiltration is higher in LRG. Antigen processing and presentation, response to IFN-Ⅰ, and cytokine related pathways also provide the basis for the immune system activation processes (Figure 7B). CNSM in LRG is significantly higher than that in HRG (Supplementary Figure S4), which indicates that the degree of genetic instability is higher. Mardis suggested that genomic instability could predict immunotherapy response more accurately. Various forms of genomic instability in cancers produce new antigens and immune-responsive phenotypes eventually (Mardis, 2019). In the analysis of LCCs and RCCs, we have confirmed that genomic instability does affect the immune response and the prognosis through a series of potential mechanisms.

Finally, in terms of clinical applications, we could significantly distinguish the sensitivity of multiple drugs by using DRPM and risk grouping. Chemotherapy has been routinely used in CC therapy, but the therapeutic effects of TIs have not been confirmed effectively (Shen et al., 2015) and a little fraction of patients benefit from immunotherapy (Lichtenstern et al., 2020). It is important to identify the patients who benefit from these treatments. We found that a variety of TIs had significant differences in sensitivity between HRG and LRG, including VEGFR inhibitor and HH inhibitors. HH has been proved to be the stemness-related signals of cancer stem cells (CSCs) (Grazioli et al., 2017; Grund-Gröschke et al., 2019). CSCs are sparks for igniting tumor recurrence and the instigators of low response to immunotherapy and drug resistance. CSCs promote the development of cancer and immunosuppression through their stemness-related signals (Lytle et al., 2018). Therefore, it has great potential to develop TIs, and the results of our research provide references for their application in CC. Meanwhile, we found that the gene expression of immunosuppressive checkpoints, T cell infiltration, and immune-related pathways was significantly enriched in LRG. In cancers with overactive T cell regulatory pathways, immune checkpoint inhibitors have been shown to be an effective strategy to enhance the anti-tumor effect and clinical impact of T cell (Hargadon et al., 2018). Therefore, we speculate that patients in the low-risk group could benefit from immunotherapy. However, the effect of immunotherapy in patients with colon cancer still needs to be further investigated.

The main limitations are as follows: firstly, while using GEO data for the verification of key lncRNAs predictive effects, two key lncRNAs were missed. Although the classification effect was propitious, the verification was not sufficient. In this regard, we examined the prognosis and clinical characteristics of all key lncRNAs to support the evidence. Secondly, we defined a mutator hypothesis-derived calculation method to evaluate genomic instability. An in-depth study is required to corroborate the functionality and significance of this method.

## Conclusion

We constructed DRPM based on the DGIA lncRNAs of LCCs and RCCs. Apart from using DRPM to predict the prognosis, we also deeply investigated the effect and mechanism of genetic instability on immunity. Meanwhile, six key DGIA lncRNAs were identified. They can not only predict the prognostic risk of patients but also serve as biomarkers for evaluating the differences of genetic instability and immune infiltration. The findings of our research can provide some basis for identifying the sensitivities of multiple treatments through genetic instability.

## Author’s Note

This is the pre-peer-reviewed version of the article: Junnan Guo, Tianyi Xia, Shenhui Deng et al. Differential Genomic Instability-Associated LncRNAs Predict Differences of Clinical Outcome and Immunity in Left- And Right- Sided Colon Adenocarcinoma, February 04, 2021, PREPRINT (Version 1) available at Research Square [https://doi.org/10.21203/rs.3.rs-175488/v1] (Junnan et al., 2021), which has been published in final form at Research Square. We have confirmed the text is our deposition as pre-print of this manuscript at Research Square (https://www.researchsquare.com/article/rs-175488/v1). This article may be used for non-commercial purposes in accordance with Frontiers Terms and Conditions for Use of Self-Archived Versions.

## Data Availability

Publicly available datasets were analyzed in this study. This data can be found here: https://portal.gdc.cancer.gov and https://www.ncbi.nlm.nih.gov/gds/.

## Supplementary Material

Supplementary Figure S1 Overall flowchart of this study.

Supplementary Figure S2 **(A)** In order to achieve a scale-free co-expression network, we chose power index = 3 as the appropriate soft threshold. **(B)** Identification of a co-expression module. The branches of the dendrogram correspond to 6 different gene modules.

Supplementary Figure S3 Kaplan-Meier curves of HRG and LRG were plotted through stratification of different clinical factors. HRG and LRG, respectively.

Supplementary Figure S4 The differences of CNSMs between HRG and LRG.
