# Supplementary figures and images for: Prognostic Immunity and Therapeutic Sensitivity Analyses Based on Differential Genomic Instability-Associated LncRNAs in Left- and Right-Sided Colon Adenocarcinoma
Source: Front Mol Biosci. 2021 Aug 31;8:668888. doi: 10.3389/fmolb.2021.668888 (PMC8438528; doi:10.3389/fmolb.2021.668888)

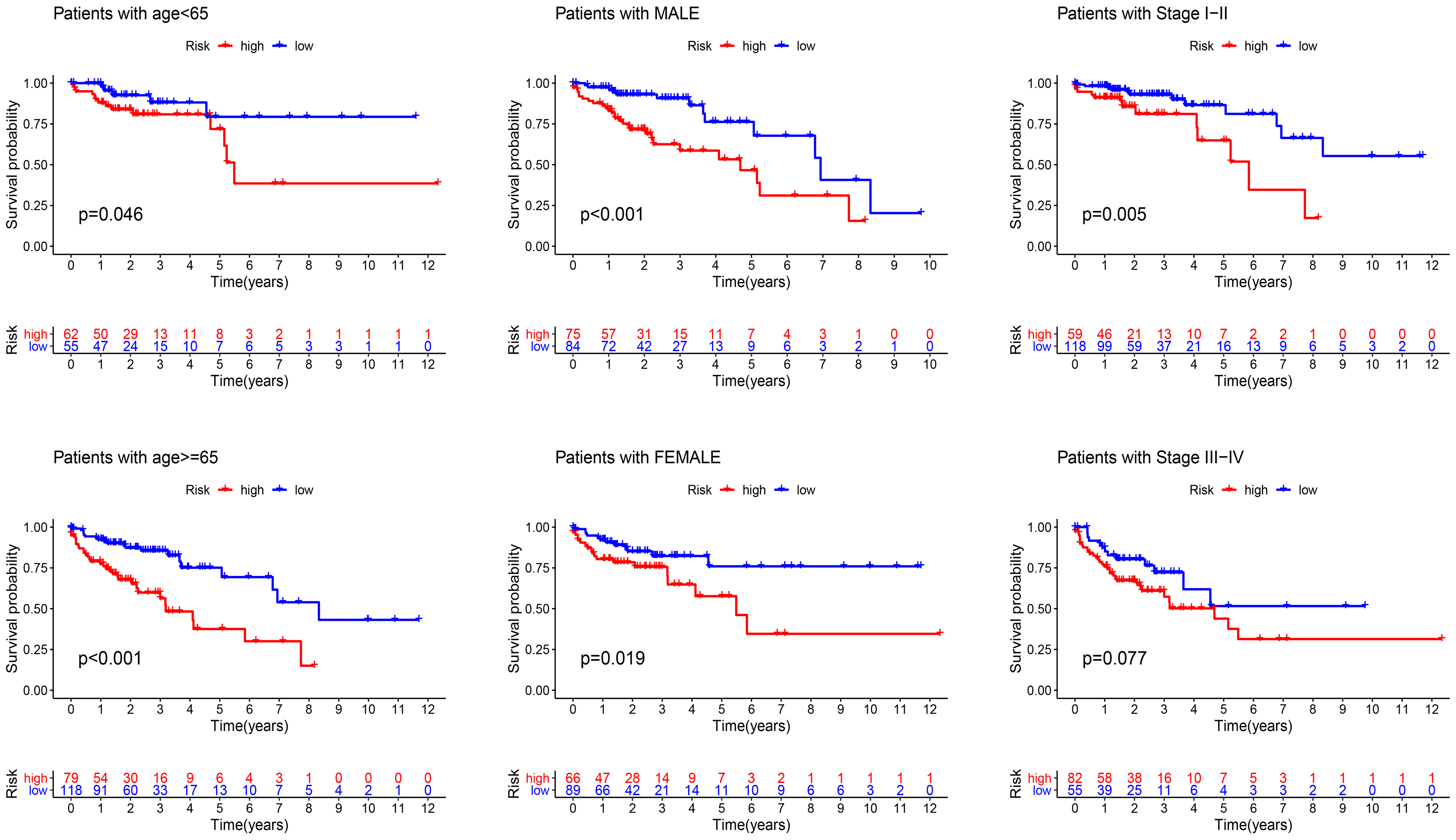

Supplement: Supplementary file 1 [file Image3.TIF]

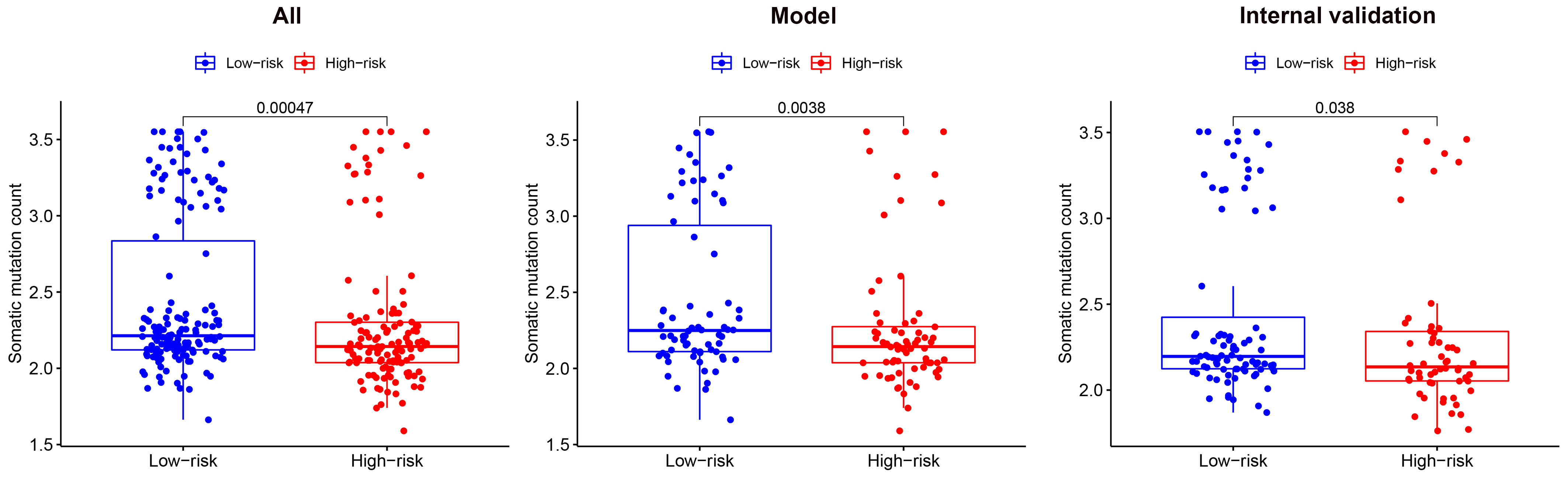

Supplement: Supplementary file 2 [file Image4.TIF]

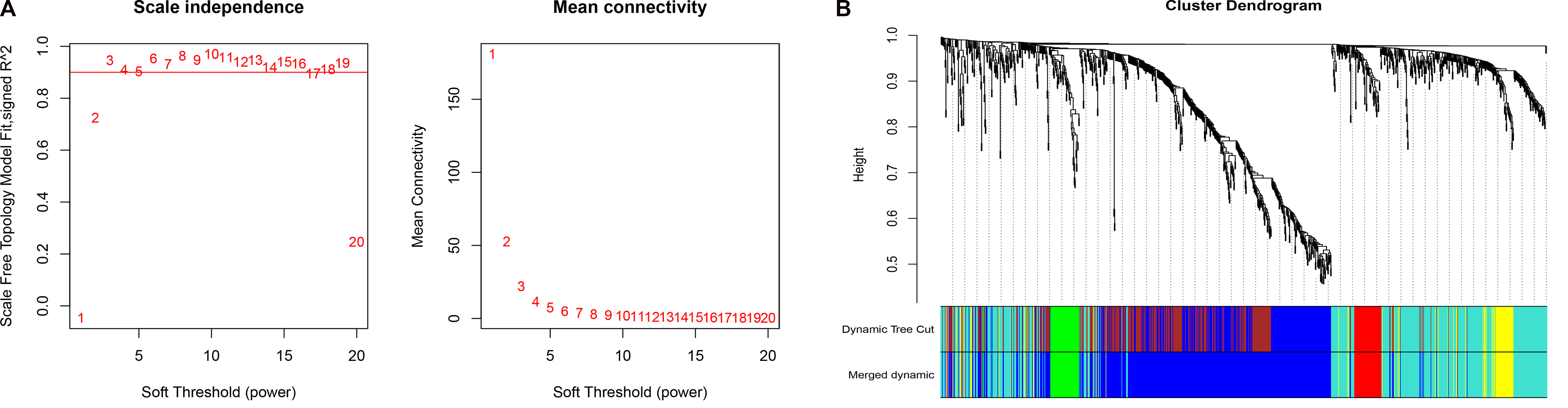

Supplement: Supplementary file 3 [file Image2.TIF]

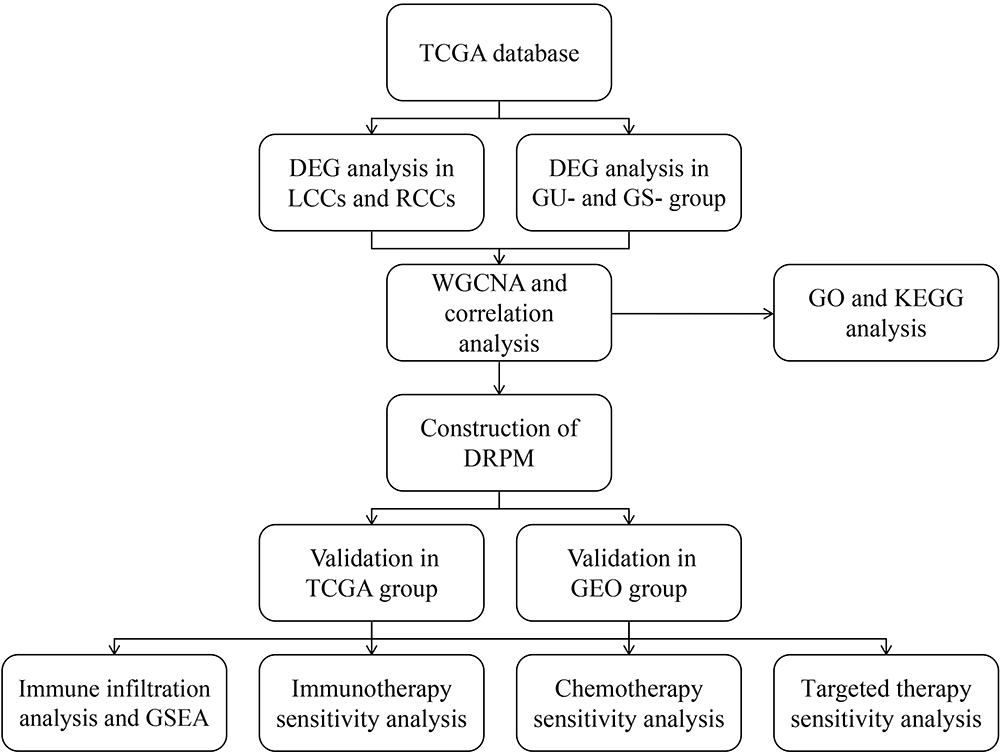

Supplement: Supplementary file 4 [file Image1.TIF]
