# Supplementary material for: Prognostic Immunity and Therapeutic Sensitivity Analyses Based on Differential Genomic Instability-Associated LncRNAs in Left- and Right-Sided Colon Adenocarcinoma
Source: Front Mol Biosci. 2021 Aug 31;8:668888. doi: 10.3389/fmolb.2021.668888 (PMC8438528; doi:10.3389/fmolb.2021.668888)
Supplement: Supplementary file 5 [file DataSheet1.docx]

Table.S1. The differences in clinical factors between model group and internal validation group.

| **Factors** | **Type** | **Total** | **Train** | **Test** | **Pvalue** |
| --- | --- | --- | --- | --- | --- |
| age | <65 | 113(36.45%) | 56(36.13%) | 57(36.77%) | 1 |
|  | >=65 | 197(63.55%) | 99(63.87%) | 98(63.23%) |  |
| gender | FEMALE | 153(49.35%) | 76(49.03%) | 77(49.68%) | 1 |
|  | MALE | 157(50.65%) | 79(50.97%) | 78(50.32%) |  |
| T | I-II | 58(18.71%) | 29(18.71%) | 29(18.71%) | 1 |
|  | III-IV | 252(81.29%) | 126(81.29%) | 126(81.29%) |  |
| N | N0 | 180(58.06%) | 86(55.48%) | 94(60.65%) | 0.4204 |
|  | N1-2 | 130(41.94%) | 69(44.52%) | 61(39.35%) |  |
| M | M0 | 262(84.52%) | 130(83.87%) | 132(85.16%) | 0.8752 |
|  | M1 | 48(15.48%) | 25(16.13%) | 23(14.84%) |  |

Table.S2. Risk clustering of patients in model and internal validation groups.

| **Id** | **Groups** | **Risk score** | **Risk clustering** |
| --- | --- | --- | --- |
| TCGA-AA-A01K | Model | 1.350001 | High |
| TCGA-AA-3979 | Model | 2.230419 | High |
| TCGA-F4-6809 | Model | 0.695534 | Low |
| TCGA-A6-A567 | Model | 1.771353 | High |
| TCGA-A6-A56B | Model | 1.933222 | High |
| TCGA-F4-6459 | Model | 8.340726 | High |
| TCGA-4T-AA8H | Model | 0.464754 | Low |
| TCGA-AA-A02H | Model | 22.78943 | High |
| TCGA-D5-6532 | Model | 0.409452 | Low |
| TCGA-AA-3655 | Model | 0.876996 | High |
| TCGA-D5-6927 | Model | 0.63495 | Low |
| TCGA-AA-A024 | Model | 0.566675 | Low |
| TCGA-G4-6315 | Model | 0.574676 | Low |
| TCGA-G4-6322 | Model | 0.862777 | High |
| TCGA-AA-3556 | Model | 1.122693 | High |
| TCGA-F4-6704 | Model | 0.984678 | High |
| TCGA-G4-6307 | Model | 0.821209 | Low |
| TCGA-AA-A00O | Model | 1.176326 | High |
| TCGA-AY-4071 | Model | 0.906942 | High |
| TCGA-D5-6924 | Model | 0.641782 | Low |
| TCGA-D5-5541 | Model | 2.577862 | High |
| TCGA-F4-6808 | Model | 1.975816 | High |
| TCGA-CM-6679 | Model | 1.162605 | High |
| TCGA-D5-6926 | Model | 1.256225 | High |
| TCGA-AY-A8YK | Model | 0.865042 | High |
| TCGA-AA-3952 | Model | 1.517018 | High |
| TCGA-CM-5868 | Model | 1.668048 | High |
| TCGA-AA-3524 | Model | 0.908884 | High |
| TCGA-A6-5662 | Model | 1.179808 | High |
| TCGA-AZ-6608 | Model | 5.57729 | High |
| TCGA-A6-6142 | Model | 2.030527 | High |
| TCGA-G4-6309 | Model | 1.05386 | High |
| TCGA-AU-3779 | Model | 0.961661 | High |
| TCGA-AA-3678 | Model | 1.154515 | High |
| TCGA-CM-6170 | Model | 0.646875 | Low |
| TCGA-AA-3529 | Model | 11.26483 | High |
| TCGA-AZ-6607 | Model | 5.647128 | High |
| TCGA-AA-A01I | Model | 0.424091 | Low |
| TCGA-AA-A02J | Model | 0.894249 | High |
| TCGA-AZ-6601 | Model | 0.68894 | Low |
| TCGA-AA-3531 | Model | 0.794273 | Low |
| TCGA-AA-3538 | Model | 1.239553 | High |
| TCGA-DM-A1D6 | Model | 1.699421 | High |
| TCGA-NH-A6GC | Model | 1.342924 | High |
| TCGA-AA-3848 | Model | 0.50367 | Low |
| TCGA-CK-4947 | Model | 0.629559 | Low |
| TCGA-CM-5344 | Model | 1.443632 | High |
| TCGA-F4-6460 | Model | 0.745383 | Low |
| TCGA-AZ-6603 | Model | 0.935873 | High |
| TCGA-G4-6299 | Model | 1.196051 | High |
| TCGA-AA-3696 | Model | 0.44562 | Low |
| TCGA-AA-3526 | Model | 1.380293 | High |
| TCGA-AA-3542 | Model | 0.98266 | High |
| TCGA-AA-A004 | Model | 0.543662 | Low |
| TCGA-AA-3972 | Model | 1.045284 | High |
| TCGA-AZ-4313 | Model | 0.475706 | Low |
| TCGA-A6-6140 | Model | 0.870797 | High |
| TCGA-AA-A01S | Model | 0.501384 | Low |
| TCGA-NH-A50T | Model | 2.528019 | High |
| TCGA-AA-3509 | Model | 0.881553 | High |
| TCGA-CA-6719 | Model | 0.968058 | High |
| TCGA-AA-A00Z | Model | 0.871357 | High |
| TCGA-AA-3977 | Model | 0.831192 | Low |
| TCGA-CK-5914 | Model | 0.791268 | Low |
| TCGA-CM-6165 | Model | 0.567414 | Low |
| TCGA-AA-A01T | Model | 0.738938 | Low |
| TCGA-QG-A5YV | Model | 0.939758 | High |
| TCGA-D5-6920 | Model | 0.445636 | Low |
| TCGA-A6-6650 | Model | 1.028858 | High |
| TCGA-CM-5349 | Model | 0.478917 | Low |
| TCGA-G4-6321 | Model | 0.497432 | Low |
| TCGA-AA-3554 | Model | 0.654733 | Low |
| TCGA-5M-AAT4 | Model | 3.276523 | High |
| TCGA-CM-4744 | Model | 0.960797 | High |
| TCGA-DM-A28K | Model | 1.083213 | High |
| TCGA-4N-A93T | Model | 0.841472 | Low |
| TCGA-AZ-6605 | Model | 1.331358 | High |
| TCGA-AD-6901 | Model | 3.745293 | High |
| TCGA-QL-A97D | Model | 0.586476 | Low |
| TCGA-AA-3534 | Model | 0.517482 | Low |
| TCGA-CK-6751 | Model | 0.794331 | Low |
| TCGA-DM-A1D9 | Model | 0.479261 | Low |
| TCGA-5M-AATE | Model | 0.654281 | Low |
| TCGA-AD-6888 | Model | 1.38537 | High |
| TCGA-AY-4070 | Model | 8.013544 | High |
| TCGA-DM-A28H | Model | 0.445002 | Low |
| TCGA-AA-A00E | Model | 0.378814 | Low |
| TCGA-DM-A1HA | Model | 4.963461 | High |
| TCGA-CM-6674 | Model | 0.540646 | Low |
| TCGA-AA-3930 | Model | 1.32368 | High |
| TCGA-CM-5864 | Model | 0.555969 | Low |
| TCGA-5M-AAT6 | Model | 3.952047 | High |
| TCGA-AA-3869 | Model | 1.256141 | High |
| TCGA-AY-6386 | Model | 0.880476 | High |
| TCGA-AA-A02R | Model | 0.591297 | Low |
| TCGA-CM-4751 | Model | 3.012129 | High |
| TCGA-CM-5862 | Model | 0.722495 | Low |
| TCGA-D5-6930 | Model | 0.492352 | Low |
| TCGA-CK-5916 | Model | 0.636746 | Low |
| TCGA-AA-3845 | Model | 7.464208 | High |
| TCGA-G4-6627 | Model | 0.557799 | Low |
| TCGA-AD-6963 | Model | 1.577316 | High |
| TCGA-AZ-4323 | Model | 1.578928 | High |
| TCGA-DM-A280 | Model | 4.479234 | High |
| TCGA-DM-A285 | Model | 2.408499 | High |
| TCGA-DM-A0X9 | Model | 0.485474 | Low |
| TCGA-AA-3496 | Model | 0.711766 | Low |
| TCGA-G4-6306 | Model | 0.681985 | Low |
| TCGA-AA-A00U | Model | 0.644304 | Low |
| TCGA-AD-6965 | Model | 1.356537 | High |
| TCGA-AD-6964 | Model | 0.685268 | Low |
| TCGA-A6-6649 | Model | 0.997365 | High |
| TCGA-DM-A288 | Model | 2.48138 | High |
| TCGA-CM-6169 | Model | 0.732541 | Low |
| TCGA-AA-3815 | Model | 0.38967 | Low |
| TCGA-AA-3862 | Model | 0.798068 | Low |
| TCGA-WS-AB45 | Model | 0.538116 | Low |
| TCGA-D5-7000 | Model | 0.52554 | Low |
| TCGA-AA-3956 | Model | 0.652664 | Low |
| TCGA-AA-A00R | Model | 0.368792 | Low |
| TCGA-AZ-4615 | Model | 0.473913 | Low |
| TCGA-A6-2679 | Model | 0.512822 | Low |
| TCGA-D5-5540 | Model | 0.443156 | Low |
| TCGA-AA-A01Q | Model | 0.356065 | Low |
| TCGA-CM-4743 | Model | 1.75592 | High |
| TCGA-CM-6162 | Model | 0.612394 | Low |
| TCGA-CK-5912 | Model | 0.86113 | High |
| TCGA-AY-A54L | Model | 0.718197 | Low |
| TCGA-CM-5348 | Model | 2.924715 | High |
| TCGA-AY-A71X | Model | 0.943674 | High |
| TCGA-CM-6171 | Model | 0.420264 | Low |
| TCGA-CK-4950 | Model | 0.754 | Low |
| TCGA-CM-5861 | Model | 0.389815 | Low |
| TCGA-CK-6747 | Model | 0.526106 | Low |
| TCGA-AA-3522 | Model | 0.815846 | Low |
| TCGA-AU-6004 | Model | 0.550996 | Low |
| TCGA-NH-A8F8 | Model | 0.894727 | High |
| TCGA-AZ-4614 | Model | 0.706116 | Low |
| TCGA-CK-4951 | Model | 1.476335 | High |
| TCGA-AZ-6599 | Model | 4.177426 | High |
| TCGA-AD-A5EK | Model | 0.47514 | Low |
| TCGA-CK-6746 | Model | 2.932527 | High |
| TCGA-QG-A5Z2 | Model | 0.481309 | Low |
| TCGA-AA-A01G | Model | 0.521087 | Low |
| TCGA-A6-2683 | Model | 0.388865 | Low |
| TCGA-AZ-5407 | Model | 0.584039 | Low |
| TCGA-G4-6302 | Model | 0.642386 | Low |
| TCGA-D5-6538 | Model | 0.47114 | Low |
| TCGA-G4-6314 | Model | 1.017668 | High |
| TCGA-AZ-6598 | Model | 5.96318 | High |
| TCGA-CM-6680 | Model | 0.714724 | Low |
| TCGA-D5-6531 | Model | 0.440629 | Low |
| TCGA-CA-6717 | Model | 0.951903 | High |
| TCGA-AA-A01Z | Model | 0.519901 | Low |
| TCGA-A6-6137 | Model | 1.048222 | High |
| TCGA-AA-A01R | Model | 0.762209 | Low |
| TCGA-AA-A01P | Model | 8.990938 | High |
| TCGA-G4-6297 | Model | 1.93512 | High |
| TCGA-AA-3543 | Model | 0.542403 | Low |
| TCGA-AA-3864 | Model | 0.770819 | Low |
| TCGA-AA-3495 | Model | 0.672415 | Low |
| TCGA-DM-A1DA | Model | 2.458991 | High |
| TCGA-CM-4746 | Internal validation | 0.597383 | Low |
| TCGA-G4-6317 | Internal validation | 0.743793 | Low |
| TCGA-AA-3968 | Internal validation | 2.070779 | High |
| TCGA-AD-6548 | Internal validation | 0.580268 | Low |
| TCGA-AA-3693 | Internal validation | 0.600684 | Low |
| TCGA-A6-2671 | Internal validation | 0.560741 | Low |
| TCGA-AZ-5403 | Internal validation | 0.95593 | High |
| TCGA-CK-5915 | Internal validation | 0.731323 | Low |
| TCGA-D5-6923 | Internal validation | 0.987903 | High |
| TCGA-A6-6648 | Internal validation | 1.025467 | High |
| TCGA-AZ-4308 | Internal validation | 0.501075 | Low |
| TCGA-A6-A566 | Internal validation | 1.364344 | High |
| TCGA-G4-6303 | Internal validation | 1.2054 | High |
| TCGA-AA-A00W | Internal validation | 0.462059 | Low |
| TCGA-AA-A017 | Internal validation | 1.390831 | High |
| TCGA-AA-A01X | Internal validation | 0.60568 | Low |
| TCGA-CM-6678 | Internal validation | 0.607433 | Low |
| TCGA-CK-6748 | Internal validation | 2.340375 | High |
| TCGA-AA-A01F | Internal validation | 0.472608 | Low |
| TCGA-CM-6161 | Internal validation | 0.592989 | Low |
| TCGA-AA-3846 | Internal validation | 1.749831 | High |
| TCGA-DM-A0XF | Internal validation | 9.266514 | High |
| TCGA-AA-3560 | Internal validation | 0.459834 | Low |
| TCGA-CM-6676 | Internal validation | 0.71181 | Low |
| TCGA-A6-6652 | Internal validation | 0.531669 | Low |
| TCGA-DM-A1D0 | Internal validation | 0.428868 | Low |
| TCGA-A6-5656 | Internal validation | 0.692214 | Low |
| TCGA-NH-A8F7 | Internal validation | 1.192693 | High |
| TCGA-QG-A5YX | Internal validation | 0.893599 | High |
| TCGA-F4-6806 | Internal validation | 0.834722 | Low |
| TCGA-F4-6855 | Internal validation | 0.62377 | Low |
| TCGA-A6-5667 | Internal validation | 2.924722 | High |
| TCGA-AA-3812 | Internal validation | 0.964182 | High |
| TCGA-CM-6172 | Internal validation | 0.49215 | Low |
| TCGA-DM-A28C | Internal validation | 0.471354 | Low |
| TCGA-A6-2674 | Internal validation | 1.437964 | High |
| TCGA-CK-4948 | Internal validation | 6.061829 | High |
| TCGA-AA-A02F | Internal validation | 0.745744 | Low |
| TCGA-F4-6854 | Internal validation | 0.655574 | Low |
| TCGA-DM-A28M | Internal validation | 0.489806 | Low |
| TCGA-AA-A02W | Internal validation | 1.018462 | High |
| TCGA-D5-6922 | Internal validation | 0.677154 | Low |
| TCGA-AA-A00F | Internal validation | 1.236453 | High |
| TCGA-DM-A28E | Internal validation | 0.6114 | Low |
| TCGA-T9-A92H | Internal validation | 1.710166 | High |
| TCGA-A6-3807 | Internal validation | 4.064604 | High |
| TCGA-A6-2675 | Internal validation | 0.693184 | Low |
| TCGA-CM-6163 | Internal validation | 0.545628 | Low |
| TCGA-AA-3712 | Internal validation | 0.840077 | Low |
| TCGA-AA-3970 | Internal validation | 2.925162 | High |
| TCGA-AA-3984 | Internal validation | 0.923581 | High |
| TCGA-CA-6715 | Internal validation | 1.309533 | High |
| TCGA-AA-3860 | Internal validation | 2.455337 | High |
| TCGA-AA-A00K | Internal validation | 0.613053 | Low |
| TCGA-CM-5341 | Internal validation | 0.567418 | Low |
| TCGA-AA-3867 | Internal validation | 2.267143 | High |
| TCGA-AA-A00Q | Internal validation | 0.596687 | Low |
| TCGA-AA-A03J | Internal validation | 0.508441 | Low |
| TCGA-AM-5821 | Internal validation | 2.336964 | High |
| TCGA-DM-A1DB | Internal validation | 0.728162 | Low |
| TCGA-DM-A28F | Internal validation | 11.07635 | High |
| TCGA-G4-6625 | Internal validation | 0.615576 | Low |
| TCGA-DM-A1D7 | Internal validation | 0.71715 | Low |
| TCGA-CM-6164 | Internal validation | 1.542738 | High |
| TCGA-D5-6541 | Internal validation | 0.828312 | Low |
| TCGA-DM-A0XD | Internal validation | 2.202251 | High |
| TCGA-AA-3675 | Internal validation | 0.6251 | Low |
| TCGA-CM-5863 | Internal validation | 1.201731 | High |
| TCGA-AD-6895 | Internal validation | 1.213522 | High |
| TCGA-AA-A022 | Internal validation | 0.798125 | Low |
| TCGA-AA-3492 | Internal validation | 0.66814 | Low |
| TCGA-NH-A50U | Internal validation | 9.658608 | High |
| TCGA-A6-6780 | Internal validation | 0.681905 | Low |
| TCGA-AA-3664 | Internal validation | 0.566803 | Low |
| TCGA-AA-A02Y | Internal validation | 0.498502 | Low |
| TCGA-AD-6890 | Internal validation | 0.610197 | Low |
| TCGA-G4-6298 | Internal validation | 1.003115 | High |
| TCGA-AZ-4616 | Internal validation | 0.625186 | Low |
| TCGA-AA-3870 | Internal validation | 0.686679 | Low |
| TCGA-A6-5661 | Internal validation | 0.848582 | Low |
| TCGA-AA-3552 | Internal validation | 1.719631 | High |
| TCGA-AA-A00D | Internal validation | 2.629805 | High |
| TCGA-AA-3680 | Internal validation | 0.650622 | Low |
| TCGA-AZ-4315 | Internal validation | 0.654523 | Low |
| TCGA-AA-3525 | Internal validation | 0.52848 | Low |
| TCGA-AA-3663 | Internal validation | 0.411338 | Low |
| TCGA-AA-3681 | Internal validation | 0.982793 | High |
| TCGA-DM-A28G | Internal validation | 0.591077 | Low |
| TCGA-A6-5665 | Internal validation | 2.719976 | High |
| TCGA-AA-A01C | Internal validation | 2.265833 | High |
| TCGA-A6-4105 | Internal validation | 0.756718 | Low |
| TCGA-AA-3861 | Internal validation | 0.522732 | Low |
| TCGA-G4-6311 | Internal validation | 1.863972 | High |
| TCGA-F4-6856 | Internal validation | 0.494154 | Low |
| TCGA-AZ-6600 | Internal validation | 0.763504 | Low |
| TCGA-CA-5796 | Internal validation | 0.858158 | High |
| TCGA-A6-2680 | Internal validation | 0.847612 | Low |
| TCGA-D5-6540 | Internal validation | 1.005363 | High |
| TCGA-A6-4107 | Internal validation | 0.65739 | Low |
| TCGA-D5-5538 | Internal validation | 0.609318 | Low |
| TCGA-D5-5539 | Internal validation | 0.893203 | High |
| TCGA-AA-A00N | Internal validation | 0.73632 | Low |
| TCGA-G4-6588 | Internal validation | 0.52491 | Low |
| TCGA-G4-6294 | Internal validation | 0.708135 | Low |
| TCGA-QG-A5YW | Internal validation | 3.48487 | High |
| TCGA-G4-6586 | Internal validation | 0.446875 | Low |
| TCGA-AA-A02K | Internal validation | 3.024017 | High |
| TCGA-CM-6168 | Internal validation | 0.732172 | Low |
| TCGA-AD-6889 | Internal validation | 0.385573 | Low |
| TCGA-AA-A02E | Internal validation | 2.601114 | High |
| TCGA-DM-A28A | Internal validation | 1.441073 | High |
| TCGA-G4-6323 | Internal validation | 0.660603 | Low |
| TCGA-AA-3520 | Internal validation | 0.448012 | Low |
| TCGA-CM-4752 | Internal validation | 0.872677 | High |
| TCGA-DM-A282 | Internal validation | 0.727503 | Low |
| TCGA-DM-A1D4 | Internal validation | 0.884402 | High |
| TCGA-A6-6782 | Internal validation | 0.622658 | Low |
| TCGA-AA-3715 | Internal validation | 1.372008 | High |
| TCGA-SS-A7HO | Internal validation | 1.02661 | High |
| TCGA-AA-3821 | Internal validation | 0.568368 | Low |
| TCGA-AA-3684 | Internal validation | 0.472926 | Low |
| TCGA-AA-A01V | Internal validation | 0.656141 | Low |
| TCGA-G4-6295 | Internal validation | 1.139446 | High |
| TCGA-AA-A00J | Internal validation | 0.408037 | Low |
| TCGA-AA-3949 | Internal validation | 0.49871 | Low |
| TCGA-3L-AA1B | Internal validation | 0.530332 | Low |
| TCGA-CM-4747 | Internal validation | 1.11157 | High |
| TCGA-CM-6675 | Internal validation | 8.057382 | High |
| TCGA-AY-6197 | Internal validation | 0.396808 | Low |
| TCGA-AA-3875 | Internal validation | 1.40701 | High |
| TCGA-CK-5913 | Internal validation | 1.182169 | High |
| TCGA-AA-3527 | Internal validation | 0.387565 | Low |
| TCGA-AD-A5EJ | Internal validation | 1.311866 | High |
| TCGA-NH-A6GA | Internal validation | 1.047153 | High |
| TCGA-CK-4952 | Internal validation | 0.467082 | Low |
| TCGA-G4-6628 | Internal validation | 0.42743 | Low |
| TCGA-AA-A00A | Internal validation | 0.419206 | Low |
| TCGA-AY-6196 | Internal validation | 1.517228 | High |
| TCGA-G4-6626 | Internal validation | 1.570075 | High |
| TCGA-RU-A8FL | Internal validation | 0.777802 | Low |
| TCGA-AZ-6606 | Internal validation | 1.190746 | High |
| TCGA-DM-A1D8 | Internal validation | 0.448254 | Low |
| TCGA-G4-6320 | Internal validation | 0.733852 | Low |
| TCGA-CM-6677 | Internal validation | 0.572932 | Low |
| TCGA-D5-6530 | Internal validation | 0.551251 | Low |
| TCGA-CM-6167 | Internal validation | 1.015985 | High |
| TCGA-CM-6166 | Internal validation | 1.244804 | High |
| TCGA-AA-A029 | Internal validation | 0.53417 | Low |
| TCGA-AD-5900 | Internal validation | 0.483012 | Low |
| TCGA-AA-3950 | Internal validation | 0.752707 | Low |
| TCGA-D5-6928 | Internal validation | 1.203924 | High |
| TCGA-AY-5543 | Internal validation | 0.717631 | Low |
| TCGA-CM-5860 | Internal validation | 0.594636 | Low |
| TCGA-AA-A03F | Internal validation | 0.788421 | Low |
| TCGA-CA-6716 | Internal validation | 0.765504 | Low |
| TCGA-F4-6703 | Internal validation | 0.699305 | Low |
| TCGA-AA-3518 | Internal validation | 0.476295 | Low |
| TCGA-NH-A50V | Internal validation | 0.717065 | Low |
| TCGA-A6-6138 | Internal validation | 0.486955 | Low |
| TCGA-AA-3710 | Internal validation | 0.721857 | Low |
